# Extremity risk factors of sepsis for gastrointestinal endoscopy in patients with liver cirrhosis

**DOI:** 10.1186/s12876-022-02124-0

**Published:** 2022-02-09

**Authors:** Yi-Chia Chan, Chao-Long Chen, Chih-Chi Wang, Chih-Che Lin, Chee-Chien Yong, King-Wah Chiu, Keng-Liang Wu

**Affiliations:** 1 Liver Transplantation Center, and Department of Surgery, Kaohsiung Chang Gung Memorial Hospital, and Chang Gung University College of Medicine, 123 Ta-Pei Road, Niao-Sung, 83303 Kaohsiung, Taiwan; 2 Liver Transplantation Center, and Department of Internal Medicine, Division of Hepato-Gastroenterology, Kaohsiung Chang Gung Memorial Hospital, and Chang Gung University College of Medicine, Kaohsiung, Taiwan

**Keywords:** Gastrointestinal endoscopy, Sepsis, Risk factors

## Abstract

**Background:**

Liver cirrhosis is a well-known risk factor of sepsis after emergent gastrointestinal (GI) endoscopy. Elective GI endoscopy before living donor liver transplantation (LDLT), however, may also carry the septic risk among these patients.

**Methods:**

This retrospective study reviewed the medical records of 642 cirrhotic recipients who underwent GI endoscopy from 2008 to 2016. We analyzed the incidence and risk factors of post-endoscopy sepsis during 2008–2012 (experience cohort). Our protocol changed after 2013 (validation cohort) to include antibiotic prophylaxis.

**Results:**

In experience cohort, 36 cases (10.5%) of the 342 LDLT candidates experienced sepsis within 48 h after endoscopy. The sepsis rate was significantly higher in patients with hepatic decompensation than patients without (22.2% vs. 9.6% vs. 2.6% in Child C/B/A groups respectively; ×2 = 20.97, *P* < 0.001). Using multivariate logistic regression analysis, the factors related to post-endoscopy sepsis were the Child score (OR 1.46; 95% CI 1.24–1.71), Child classes B and C (OR 3.80 and 14.13; 95% CI 1.04–13.95 and 3.97–50.23, respectively), hepatic hydrothorax (OR 4.85; 95% CI 1.37–17.20), and use of antibiotic prophylaxis (OR 0.08; 95% CI 0.01–0.64). In validation cohort, antibiotics were given routinely, and all cases of hepatic hydrothorax (n = 10) were drained. Consequently, 4 (1.3%) episodes of sepsis occurred among 300 LDLT candidates, and the incidence was significantly lower than before (1.3% vs. 10.5%, *P* < 0.001).

**Conclusions:**

Patients with decompensated cirrhosis and hepatic hydrothorax have higher risk of sepsis following endoscopy. In advanced cirrhotic patients, antibiotic prophylaxis and drainage of hydrothorax may be required to prevent sepsis before elective GI endoscopy.

## Introduction

Liver cirrhosis has become one of the major causes of mortality and morbidity, and liver transplantation (LT) provides the most effective surgical treatment [1, 2]. According to the guidelines of the United Network for Organ Sharing and American Association for the Study of Liver Disease, it is mandatory that the presence of extrahepatic malignancy be excluded in LT candidates before surgery [3, 4]. The reason for exclusion comes from the Cincinnati Transplant Tumor Registry study by Penn et al. in 1997. In that study, observation data were obtained from more than 1000 renal transplant recipients with history of malignancy, which showed a 22% recurrence rate after transplantation [5]. At present, the mechanism of cancer recurrence is still unknown, but it may result from immunosuppressive therapy, including attenuation of the functions of cytotoxic T-cells and natural killer cells, as well as the disturbance of humoral interaction with macrophages [6]. Transplant recipients under immunosuppression drugs have increased risks of cancer recurrence, so surveying for extrahepatic malignancy with at least a 5-year tumor-free interval is recommended [7].

The database of the Taiwan National Health Institution indicate that in 2013, colorectal, stomach, and esophageal cancers were ranked as the first, seventh, and tenth highest priorities among all kinds of newly diagnosed malignancies in Taiwan [8]. Esophagogastroduodenoscopy (EGD) and colonofiberscopy (CFS) are the most available standard tools to detect the GI malignancy and have advantages of providing imaging surveys and biopsy proof. The current practice guidelines of the American Society of Gastrointestinal Endoscopy (ASGE) discourage antibiotic prophylaxis in cirrhotic patients without active upper GI bleeding [9], but there have been several case reports of septicemia or peritonitis after elective colonoscopy [10–12]. In addition, only two cases of septicemia following CFS were observed in a cohort of 5000 patients, but there was one 20-year-old male with advanced cirrhosis secondary to sclerosing cholangitis who was awaiting LT but died of severe sepsis [10]. This indicates that potentially fatal infection may occur if prophylactic antibiotics are not given to patients with cirrhosis undergoing endoscopy.

Hepatic hydrothorax (HH) was first defined by Morrow et al. in 1958 while describing a rapid accumulation of pleural effusion among cirrhotic patients [13]. HH frequently occurs in combination with ascites and other features of portal hypertension resulting from decompensated liver cirrhosis. Ricardo et al. found cirrhotic patients with HH had shorter life expectancies and poor outcomes than patients without HH [14].

In this study, we report our experience with sepsis associated with upper and lower GI endoscopy among cirrhotic patients. First, we determined the risk factors based on data from 2008 to 2012 (experience cohort). Next, we investigated an effective strategy to reduce the sepsis risks following endoscopy using data from 2013 to 2016 (validation cohort).

## Patients and methods

### Study population and design

**Fig. 1:**
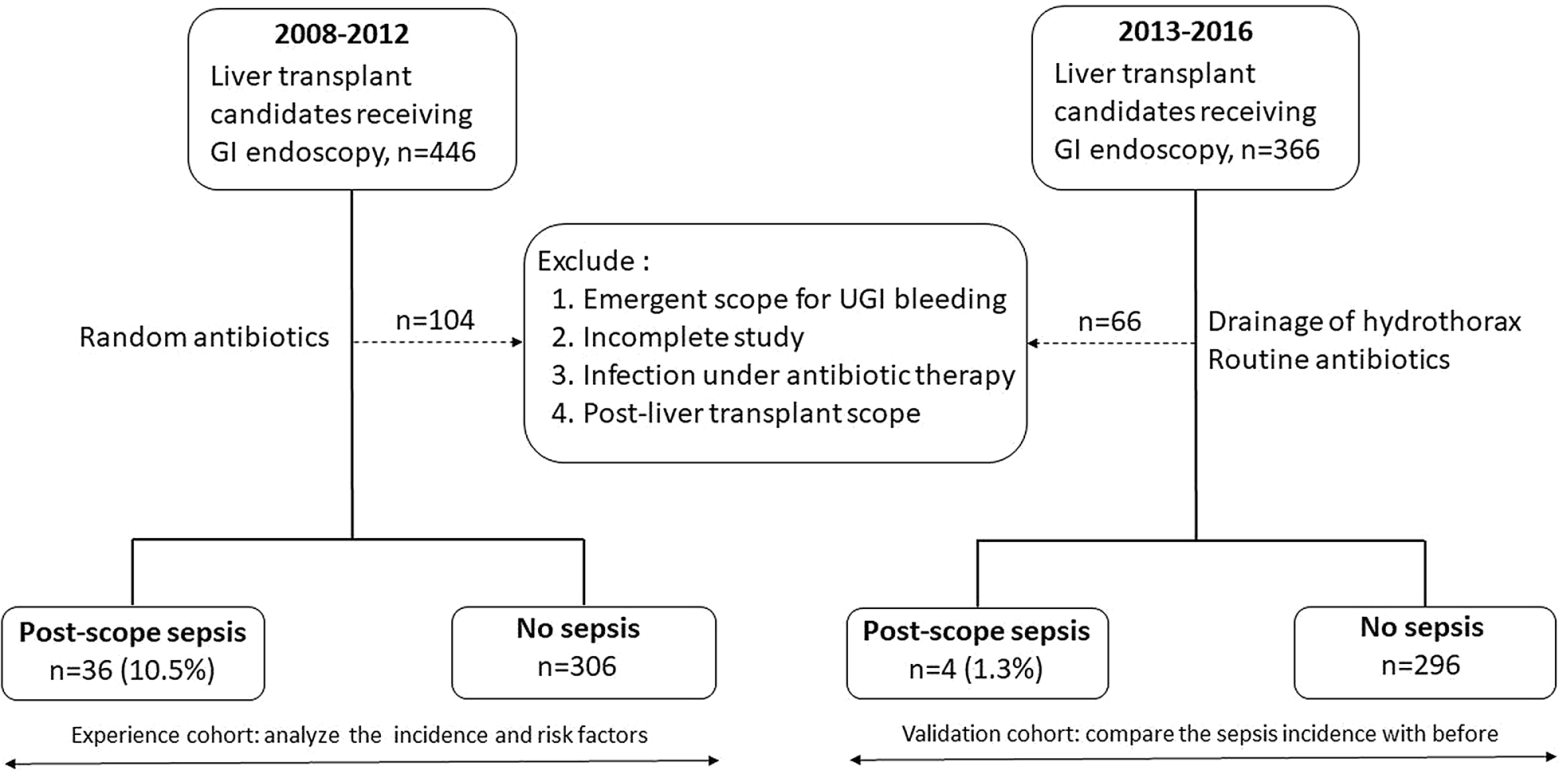
Outline of the study design. During 2008–2012 (experience cohort), we analyzed the risk factors associated with sepsis following gastrointestinal endoscopy. Since 2013 (validation cohort), prophylactic antibiotic was given for all liver transplant candidates before the endoscopy exam

Kaohsiung Chang Gung Memorial Hospital, Taiwan, maintains a longitudinal database of primary living donor LT (LDLT) recipients and records all demographic, pre-operative, peri-operative, pathological, and follow-up information. After the approval of the hospital’s institutional review board (IRB no. 201800328B0), we retrospectively reviewed the records of all adults (> 18 years old) who received LDLT between September 2008 and December 2012 (experience cohort) to determine the incidence and risk factors of sepsis associated with GI endoscopy before transplant surgery. Furthermore, we also reviewed data from January 2013 to December 2016 (validation cohort) to evaluate the effectiveness of a changes in protocol to reduce the rate of sepsis rate (Fig. 1). All the study protocol was in accordance with Declaration of Helsinki.

### Inclusion and exclusion criteria

The study included adult patients aged 18–70 years old with ESLD or hepatocellular carcinoma (HCC) who needed LT as therapy. The exclusion criteria were emergency GI endoscopy for acute GI bleeding, incomplete endoscopy exams, ongoing therapeutic antibiotic treatment for active infection, and the postponement of endoscopy after transplant surgery.

### Definitions

Sepsis is defined as a combination of more than one criteria for systemic inflammatory response syndrome (SIRS) and the presence of documented or presumed infection, which was proposed in the 1992 North American consensus document [15]. The SIRS criteria include (1) body temperature over 38 °C or under 36 °C; (2) heart rate greater than 90 beats/minute; (3) respiratory rate greater than 20 breaths/minute or partial pressure of CO_2_ less than 32 mmHg; and (4) leukocyte count greater than 12,000/microliter or less than 4000/microliter or the presence of over 10% immature forms or bands [15, 16]. Sepsis associated with GI endoscopy is defined as sepsis occurring within 72 h after EGD and CFS [17]. HH is defined as excessive accumulation of transudate (> 500 ml) in the pleural cavity in cirrhotic patients but without pleural and cardiopulmonary disease [18].

### Data collection

The demographics of recipients were collected along with pre-operative and operative variables. Demographic and clinical variables included ages, sex, and underlying etiology of liver cirrhosis, in addition to the coincident diagnosis of HCC. Pre-operative variables included incidence of diabetes mellitus (DM) and hypertension (HTN), Child–Pugh (CTP) score, model of end-stage liver disease (MELD) score, body weight, presence of ascites and HH, white blood cell count, hemoglobin, platelet count, serum albumin, total bilirubin, creatinine level, and prothrombin time (PT). Intra-operative variables included the volume of ascites evacuated at the time of abdominal incision.

### Pre-operative assessment

The pre-operative assessment included psychological examination and radiological assessment of the hepatic vasculo-biliary anatomy of both the donor and recipient with liver computed tomography (CT) angiography and magnetic resonance imaging. Chest CTs were also applied as a screening tool for asymptomatic lung nodules [19]. The decision for LDLT was made in weekly multidisciplinary meetings.

### Gastrointestinal endoscopy procedures

All liver transplant candidates received GI endoscopy at least 5 days before transplant surgery to assure the timely pathological proof of benign lesions. Bowel preparation was performed in-hospital to ensure adequate diet restriction and to provide intravenous fluid to avoid dehydration and electrolyte imbalance. On the day before endoscopy, the patients were requested to eat a low-residue diet for breakfast and lunch and a residue-free diet for dinner, followed by fasting after midnight to maintain gastric emptying at least eight hours before the endoscopy. We diluted one sachet of Niflec (Ajinomoto Pharma Co., Ltd.) containing polyethylene glycol (PEG) in 2 L of water to make a solution for two usages. The patients drank the 2 L at a rate of about 1 L per hour. If the excretion fluid was already clear, they stopped taking the drug.

Prophylactic antibiotics with third-generation cephalosporin were given at random to 41 LT candidates among the 342 patients in the experience cohort. More specifically, antibiotics was prescribed by some of senior residents who had experienced sepsis in patients undergoing GI endoscopy. These LT candidates were assigned randomly for primary care to our residents. Our regimen for antibiotic prophylaxis included one of the following: ceftriaxone at 1 g/12 h, ceftazidime at 1 g/8 h, or flomoxef at 1 g/8 h intravenously for a one-day course starting 30 min before endoscopy.

Combined EGD and CFS under intravenous general anesthesia were performed using propofol without intubation by experienced endoscopists and anesthesia doctors. Biopsy or polypectomy was performed if indicated after correction of coagulopathy or thrombocytopenia. The procedure was completed within approximately 1 h.

After the endoscopy examination, all LT candidates stayed in the hospital and were observed every four to eight hours in the regular ward. If occurrence of abdominal pain or fever or unstable vital signs, appropriated medical management was initiated, including blood tests, cultures, or image surveys following the principal recommendations [16]. Empirical antibiotics were given to sepsis patients as soon as possible to prevent disease progression.

### Changed protocol in the validation cohort (years 2013–2016)

In the validation cohort, the procedure was the same as in the experience cohort except that drainage was performed for all HH (n = 10, 3.3%), and prophylactic cephalosporin was given to all liver-transplant candidates before endoscopy.

### Statistical analysis

Data were analyzed using the software IBM SPSS version 20. Qualitative variables were expressed as numbers (percentages) and compared using the chi-squared test. Quantitative variables were expressed as the mean ± standard deviation (SD) and compared between two groups using an independent-samples *t* test if normally distributed or a Mann–Whitney *U* test if not normally distributed. Univariate analysis was performed using relevant tests to examine the association of different variables with the development of sepsis associated with GI endoscopy. Variables with a *P* value < 0.2 were entered into a multivariate analysis with stepwise logistic regression to select independent risk factors of sepsis. The results were considered significant if the *P* value was < 0.05. Receiver operating characteristic (ROC) curve was used to calculate cutoff values to determine the prognostic value of CTP score with regard to the development of sepsis associated with GI endoscopy.

## Results

### Demographic data of the experience and validation cohorts

During the study period, 812 adult patients underwent LDLT at our center. We excluded 104 and 66 patients in the experience and validation cohorts, respectively. Causes of exclusion were: emergency endoscopic hemostasis for acute GI bleeding (n = 9 and 10, respectively), active infection with ongoing antibiotic treatment (n = 50 and 10, respectively), incomplete endoscopic exams like only EGD or only CFS or incomplete study because of intolerable pain (n = 22 and 12, respectively), or postponed endoscopy examination until recovery after transplantation (n = 23 and 34, respectively). Overall, there were 342 and 300 liver transplant candidates in the respective cohorts. The study flowchart is shown in Fig. 1.

The final cohort comprised 642 patients, and their mean age was 54 years. Most of them were men (70.8%) with a median Child–Pugh score of 7.7. The majority of the patients had chronic HBV infection (n = 287, 43.9%) or HCC (n = 371, 56.7%), which was the main indication for LDLT. The patient’s demographics are shown in Tables 1 and 2.

**Table 1:** Patient characteristics and baseline variables in the experience cohort (2008–2012)

	Sepsis following EGD and CFS		*P* value
	Present (n = 36)	Absent (n = 306)	
Recipient age (year)	53.4 ± 7.3	54.6 ± 8.7	0.401
Male sex, n (%)	29 (80.6)	230 (75.2)	0.475
Body weight (kg)	70.9 ± 14.2	67.0 ± 13.3	0.225
MELD score	14.78 ± 4.66	12.11 ± 6.18	0.013
Child–Pugh score	9.53 ± 2.04	7.68 ± 2.37	< 0.001
Child–Pugh classification, n (%)			< 0.001
A	3 (8.3)	113 (36.9)	
B	13 (36.1)	123 (40.2)	
C	20 (55.6)	70 (22.9)	
Diabetes mellitus, n (%)	5 (13.9)	56 (18.3)	0.513
Hypertension, n (%)	4 (11.1)	23 (7.5)	0.449
Primary liver disease, n (%)			
HBV	17 (42.7)	143 (46.7)	0.955
HCV	15 (41.7)	109 (35.6)	0.475
Alcoholic	2 (5.6)	21 (6.9)	0.767
HCC positive	15 (41.7)	192 (62.7)	0.014
Ascites* (ml)	550 (3375)	0 (750)	0.180
Ascites* > 1L, n (%)	18 (50.0%)	75 (24.5%)	0.001
Hepatic hydrothorax^#^, n (%)	5 (13.9%)	8 (2.6%)	0.001
Prophylactic antibiotic, n (%)	1 (2.8%)	42 (13.7%)	0.061
Laboratory			
WBC (× 10^9^/L)	3.7 ± 2.5	4.2 ± 2.9	0.288
Platelet (10^9^/L)	49.0 ± 25.3	64.4 ± 42.7	0.002
Albumin (g/dL)	2.7 ± 0.5	3.2 ± 0.6	< 0.001
Total bilirubin (mg/dL)	2.1 (2.5)	1.5 (1.4)	< 0.001
Creatinine (mg/dL)	0.8 ± 0.5	0.8 ± 0.3	0.430
Prothrombin time (second)	14.4 ± 2.4	12.8 ± 2.4	< 0.001

Data are shown as the mean ± standard deviation, number (%), and median (interquartile range (IQR) 25–75) unless otherwise stated

*Evacuated and estimated at time of abdominal incision

#Hepatic hydrothorax: pleural effusion > 500 cc

MELD: Model for End stage Liver Disease

**Table 2:** Patient characteristics and baseline variables between the 2 cohorts

	Year 2008–2012 Experience cohort (n = 342)	Year 2013–2016 Validation cohort (n = 300)	*P* value
Recipient age	54.5 ± 8.5	54.3 ± 8.6	0.848
Male sex, n (%)	259 (75.7)	204 (68.0)	0.029
Body weight (kg)	66.8 ± 11.7	67.5 ± 12.2	0.490
MELD score	12.4 ± 6.1	11.9 ± 5.6	0.344
Child–Pugh score	7.8 ± 2.4	7.6 ± 2.3	0.210
Child–Pugh classification, n (%)			0.444
A	116 (33.9)	115 (38.3)	
B	136 (39.8)	116 (38.7)	
C	90 (26.3)	69 (23.0)	
Primary liver disease, n (%)			
HBV	160 (46.8)	126 (42.0)	0.224
HCV	124 (36.3)	97 (32.3)	0.296
Alcoholic	23 (6.7)	50 (16.7)	< 0.001
HCC positive	207 (60.5)	164 (54.7)	0.134
Ascites* > 1L, n (%)	93 (27.2)	93 (31.0)	0.289
Hydrothorax, n (%)	13 (3.8)	10 (3.3)	0.750
Prophylactic antibiotic, n (%)	43 (12.6)	300 (100)	< 0.001
Sepsis	36 (10.5)	4 (1.3)	< 0.001

Data are shown as the mean ± standard deviation, number (%), and median (IQR) unless otherwise specified

*Evacuated and estimated at time of abdominal incision

MELD: Model for End stage Liver Disease

### Incidence and clinical presentations of GI-endoscopy-associated sepsis (experience cohort)

**Fig. 2:**
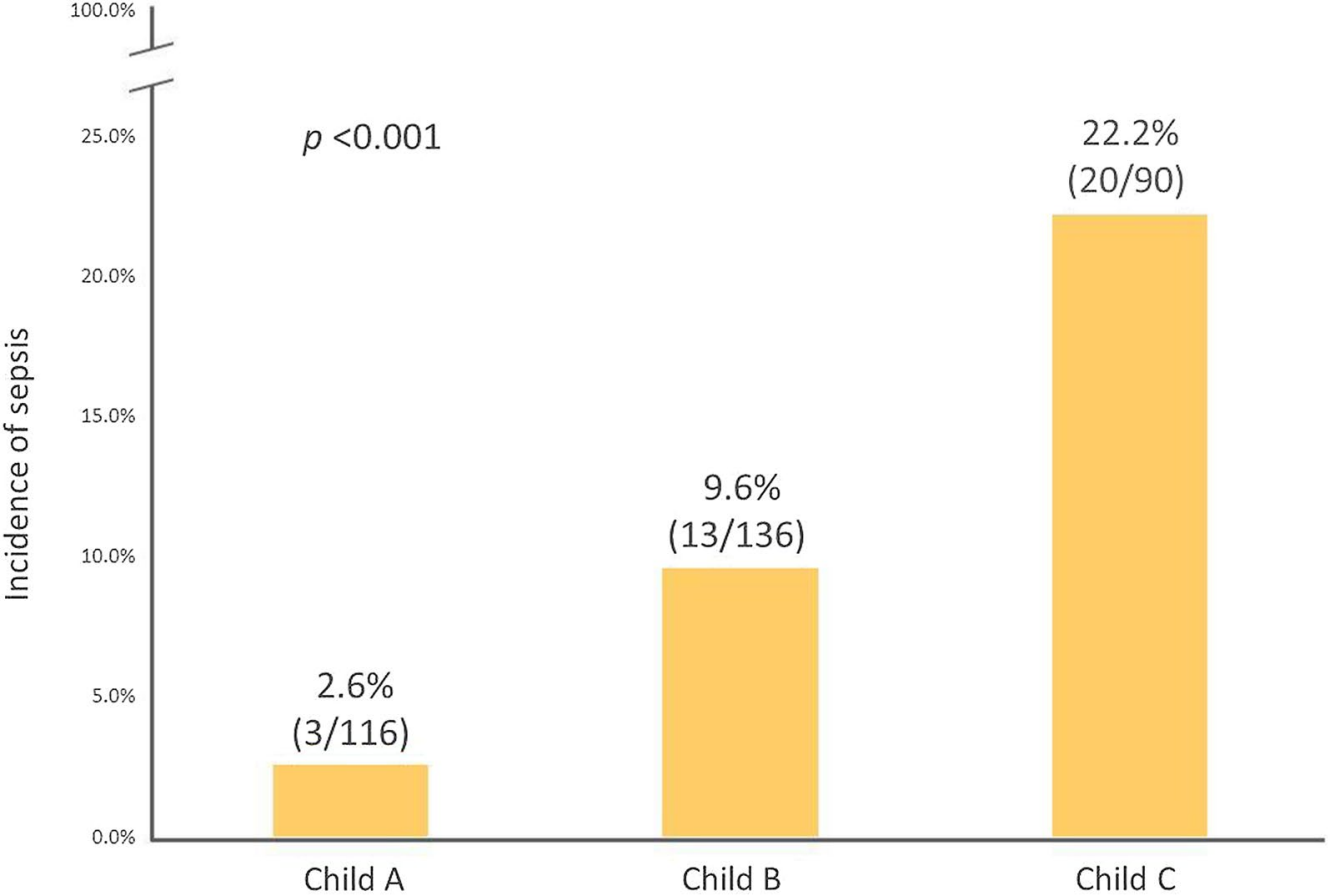
Receiver operator characteristic (ROC) curve analysis of Child–Pugh score to predict the occurrence of sepsis after GI endoscopy

There were 342 LT candidates in the first cohort (Table 1). The incidence of post-endoscopy sepsis was 10.5% (36/342), and higher probability was associated with the severity of Child–Pugh classifications (x^2^ = 20.97, *P* < 0.001) (Fig. 2). The timing of sepsis onset was within 24 h after endoscopy in 26 cases (72%) and 24–48 h in 10 cases (28%). No endoscopy-associated sepsis occurred after 48 h.

Among the 36 cases of sepsis, the most common presentation of SIRS was fever more than 38 °C, which occurred in 33 patients (92%), who had an average temperature of 38.8 ± 0.7 °C. The second most common presentation was tachypnea in 21 patients (58%) and accelerated heart rate in 21 patients (58%) (Table 3). Notably, there were four patients who had confirmed bacteremia, although blood culture was yielded in every case. The four positive blood cultures showed *Micrococcus* (n = 1), *Staphylococcus aureus* (n = 1), *Escherichia coli* (n = 1), and *Aeromonas sobria* (n = 1) bacteremia.

**Table 3:** Clinical presentations of 36 patients with sepsis following EGD and CFS in the experience cohort (year 2008–2012)

Characteristic	N (%)
Onset of sepsis after scope	
26 (72%)	< 24 h
10 (28%)	24-48 h
Fever > 38˚C	33 (92%)
Body temperature* (˚C)	38.8 ± 0.7 (38.1–40.4)
Respiratory rate > 20 breaths/min	21 (58%)
Pulse rate > 90 beats/min	21 (58%)
Abdominal pain	9 (25%)
Bacteremia	4 (11%)
Peritonitis	2 (6%)
Septic shock	1 (3%)

Data are shown as number (%)

*Average ± SD and (minimal to maximal) body temperature of 33 patients with fever

Another patient experienced septic shock with concomitant peritonitis less than 24 h after the GI endoscopic procedure without biopsy or polypectomy, but the patient recovered well three days later after prompt antibiotic treatment. There were five septic cases combined with HH, but none of them presented pulmonary symptoms before or after endoscopic procedures. In the pleural fluid analysis, transudate was tested using Light’s criteria and no bacteria growth in cultures.

### Predictors of sepsis associated with GI endoscopy in the experience cohort (Year 2008–2012)

**Fig. 3:**
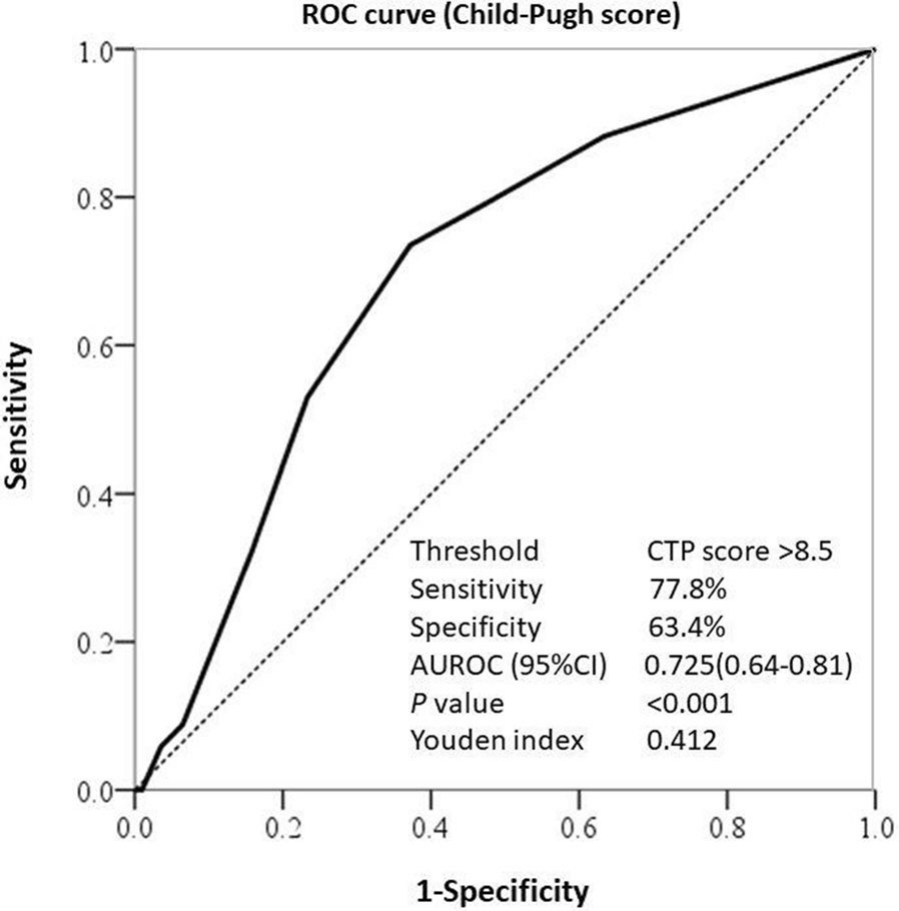
Prevalence of post-endoscopic sepsis according to the Child–Pugh class in cirrhotic patients (*x*^2^ = 20.97, *P* < 0.001) in the experience cohort, which shows significant increases

A comparison between the sepsis and non-sepsis groups is shown in Table 1. A binomial logistic regression was performed to determine the risk factors related to GI-endoscopy-associated sepsis. In the univariate analysis, several variables were significantly correlated with sepsis: the presence of HCC, MELD score, CTP score, Child–Pugh B and C cirrhosis, more than 1 L of ascites, HH, serum platelet count, albumin, bilirubin level, and PT. In the multivariate analysis, only the CTP score, Child–Pugh B and C classification, HH, and prophylactic antibiotic use reached *P* values of < 0.05 (Table 4). A CTP score cutoff value of > 8.5 was calculated as a predictor of post-GI endoscopy sepsis by the ROC curve analysis (Fig. 3). The AUC was 0.725, and the threshold CTP score of 8.5 reached a sensitivity of 77.8% and a specificity of 63.4%.

**Table 4:** Logistic regression analysis for risk factors of GI-endoscopy-associated sepsis

Variables	Univariate analysis		Multivariate analysis	
	OR (95% CI)	*P*	OR (95% CI)	*P*
Age	0.98 (0.95–1.02)	0.400		
Male sex	1.37 (0.58–3.26)	0.477		
Body weight	1.02 (0.99–1.05)	0.226		
Diabetes mellitus	0.72 (0.27–1.93)	0.515		
Hypertension	1.54 (0.50–4.73)	0.452		
HCC	0.42 (0.21–0.86)	0.017		
MELD score	1.06 (1.01–1.11)	0.016		
Child–Pugh score	1.37 (1.18–1.59)	< 0.001	1.46 (1.24–1.71)	< 0.001
Child–Pugh classification A B C	1 3.98 (1.11–14.33) 10.76 (3.09–37.55)	0.035 < 0.001	1 3.80 (1.04–13.95) 14.13 (3.97–50.23)	0.044 < 0.001
Ascites > 1L	3.08 (1.52–6.22)	0.002		
Hepatic hydrothorax	6.01 (1.85–19.49)	0.003	4.85 (1.37–17.20)	0.014
Prophylactic antibiotic	0.18 (0.02–1.35)	0.095	0.08 (0.01–0.64)	0.017
Platelet (10^9^/L)	0.99 (0.98–1.00)	0.037		
Albumin (g/dL)	0.30 (0.16–0.57)	< 0.001		
Total bilirubin (mg/dL)	1.08 (1.03–1.12)	0.002		
Prothrombin time (second)	1.22 (1.09–1.38)	0.001		

MELD: Model for End stage Liver Disease

### Association between biopsy and polypectomy with sepsis in the experience cohort

The invasive part of the endoscopic procedure consisted of biopsy or polypectomy of gastric and colon lesions. Gastroduodenal biopsy or polypectomy was performed in 41.7% and 36.6% of the sepsis and non-sepsis groups (*P* value = 0.552), and colon biopsy or polypectomy was performed in 55.6% and 49.7% of the groups, respectively (*P* value = 0.504), but the differences were not significant (Table 5).

**Table 5:** Relationship of endoscopic procedure and post-scope sepsis

	Sepsis following EGD and CFS		*P* value
	Present (n = 36)	Absent (n = 306)	
Esophagogastroduodenoscopy (EGD), n (%)			
Biopsy	14 (38.9)	104 (34.0)	0.558
Polypectomy	2 (5.6)	5 (1.6)	0.116
Either/both biopsy or polypectomy	15 (41.7)	112 (36.6)	0.552
Colonofiberscopy (CFS), n (%)			
Biopsy	15 (41.7)	103 (33.7%)	0.339
Polypectomy	7 (19.4)	54 (17.6)	0.790
Either/both biopsy or polypectomy	20 (55.6)	152 (49.7)	0.504

Data are shown as number (%)

### Validation cohort (years 2013–2016) routine antibiotic prophylaxis and drainage of hepatic hydrothorax

There were four cases of sepsis following GI endoscopy in the validation cohort. One of them had tachycardia and low-grade fever before endoscopy, and occult or ongoing infection was suspected before the procedure. The remaining three cases had higher CTP scores (8, 9, and 10, respectively) than the mean CTP score of 7.6 in this cohort (Table 2), and no case was diagnosed with bacteremia.

### Comparison between the experience and validation cohorts

**Fig. 4:**
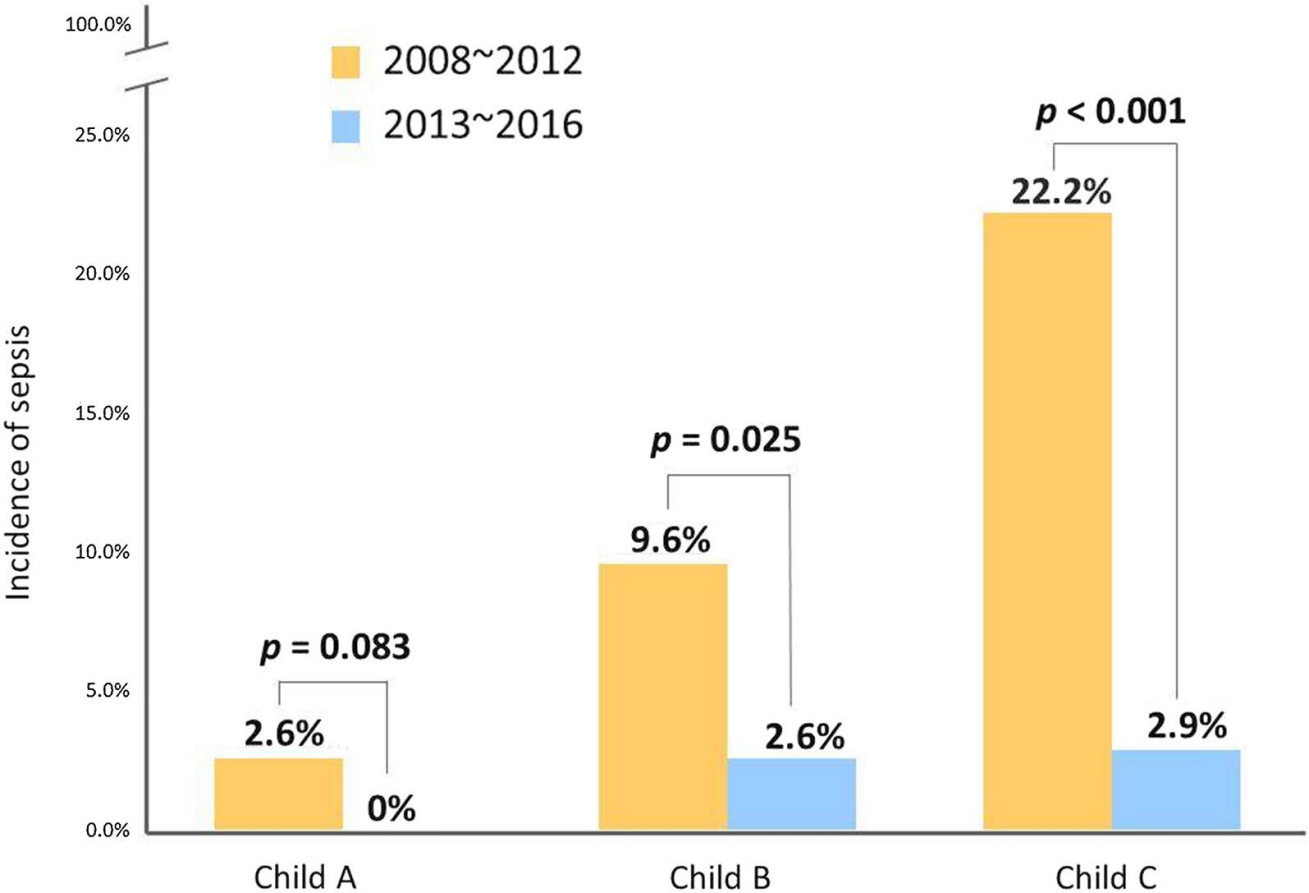
Decreased incidence of GI-endoscopy-associated sepsis in cirrhotic patients according to severity of Child–Pugh classification after routine prophylactic antibiotic and drainage of hepatic hydrothorax after 2013 (validation cohort)

The demographics of the validation cohort were comparable with those of the experience cohort except for a proportion of females and alcoholic liver cirrhosis (Table 2), but neither of these was a risk factor for sepsis. The revised protocol including drainage of all HH (10 patients (3.3%)) and overall antibiotic prophylaxis in every case prior to the endoscopy procedure made the sepsis rate decrease to 1.3% (4 cases), which is significantly lower than in the experience cohort (10.5%; *P* < 0.001; Table 2^2^). Moreover, the improvement of sepsis prevention for GI endoscopy was also demonstrated in the decompensated cirrhotic groups (Child–Pugh B: 9.6% vs. 2.6%; *P* = 0.025; Child–Pugh C: 22.2% vs. 2.9%; *P* < 0.001 in the experience and validation cohorts, respectively; Fig. 4).

## Discussion

We observed a sepsis rate of 10.5% after GI endoscopy in cirrhotic patients waiting for LT. Decompensated cirrhosis (Child–Pugh B and C classifications) and HH were important risk factors in the experience cohort. Drainage of HH and routine antibiotic prophylaxis before endoscopic procedures in the validation cohort reduced the incidence of sepsis to 1.3% with significant improvement regarding the Child–Pugh B and C categories. Bacterial infections are diagnosed in 40% of in-hospital cirrhotic patients and lead to a four-fold increase in mortality [20]. The literature also pointed out that sepsis and bacterial infection are recognized as distinct stages in the progression of chronic liver disease that speed up the organ failure and contribute to the high mortality [20].

We used the definition of sepsis from the criteria of SIRS for documented or suspected infection [15, 16], which has high sensitivity and low specificity compared to the new sepsis definition based on organ dysfunction using Sequential Organ Function Assessment (SOFA) [21]. Nevertheless, Julian et al. reported that SIRS is associated with mortality and organ failure with relative risk of 3.2 and 3.5, respectively, and that it is not inferior to the SOFA criteria. These results were obtained in a large prospective study comprising 8871 patients [21]. Moreover, even in the absence of bacterial infection, the occurrence of SIRS in cirrhotic patients has prognostic relevance, which might aggravate portal hypertension, renal failure, and hepatic encephalopathy, thereby contributing to multi-organ failure and mortality [22]. Therefore, the high sensitivity of SIRS has fundamental clinical importance for identifying sepsis early and applying effective treatment to minimize complications in cirrhotic patients who have high infection risk.

The severity of liver disease is associated with increased risks of bacterial infection and correlated with mortality after infection [23]. Numerous variables were associated with mortality after bacterial infection in a meta-analysis by Arvaniti et al. [23]. There were five variables related to the severity of cirrhosis (CTP score, PT, bilirubin, albumin, and MELD score) and three related to cirrhosis-associated complications (encephalopathy, GI hemorrhage, and HCC). Similar to their findings, several parameters in our study associated with liver function were found to be relevant to post-endoscopy sepsis in the univariate analysis (HCC, MELD score, Child score, Child–Pugh classification, ascites, HH, platelet count, PT, and serum albumin and bilirubin levels). However, only the CTP score, Child–Pugh classes B and C, HH, and no use of prophylactic antibiotics were risk factors for sepsis in the multivariate analysis (Table 4).

The MELD score consists of three variables: serum International Normalized Ratio (INR), total bilirubin level, and creatinine level. However, the CTP score is calculated using five variables: ascites, encephalopathy, PT, and serum levels of bilirubin and albumin. The CTP score uses more clinically significant parameters (albumin, bilirubin, PT, and ascites) than the MELD score (bilirubin, INR) to predict the endoscopy-associated sepsis, which makes using the CTP score for prediction more favorable. Moreover, our data showed that decompensated cirrhosis (Child–Pugh B and C) is more predisposing for sepsis compared with Child–Pugh A compensated liver cirrhosis, and the cutoff point of the CTP score was over 8.5 (Fig. 3), which also corresponds to Child–Pugh classes B and C.

Cirera et al. reported that the prevalence of bacterial translocation significantly increased according to the Child–Pugh classification, with 3.4% for Class A, 8.1% for Class B, and 30.8% for Class C patients (*x*^2^ = 6.106, *P* < 0.05). This may explain the occurrence of sepsis or bacteremia following GI endoscopy correlating with the severity of cirrhosis in our results [24]. Another two variables, platelet count and HCC, also reflect the complexity of liver cirrhosis. Thrombocytopenia is a marked feature of chronic liver disease and cirrhosis, especially in patients with hepatitis B and C infections compared to other causes of chronic liver disease [25]. In our cohort, the LT candidates who also had HCC had less severe liver dysfunction than other patients without HCC (CTP score in HCC group: 7.0; non-HCC group: 9.2; *p* < 0.001). This may explain the presence of HCC being associated with lower risk of infection.

In our study, the occurrence of HH was 3.6% (23/642) during 2008 to 2016, which is slightly lower than the incidence rate of 5–10% reported by other studies [18]. This difference could have occurred because our patients were LT candidates, including HCC groups without portal hypertension or cirrhotic change. In the total of 23 cases presenting with HH, only 11 patients (47.8%) also had massive ascites greater than one liter and showed poor response to diuretics. In the univariate and multivariate analyses, HH was a significant risk predisposing factor for sepsis associated with GI endoscopy (OR = 4.69, *P* = 0.016), but none of our patients presented with pulmonary symptoms or empyema or pneumonia.

In the experience cohort, HH was not drained in all patients, and the sepsis rate was 38.4% (5/13). However, in the validation cohort, HH was recognized as a risk factor for post-endoscopy sepsis and poor response to diuretics and fluid restriction. Thus, all patients with HH received antibiotic prophylaxis and chest pigtail drainage prior to endoscopy procedures, so no patient experienced sepsis. In the 77 cases of HH reviewed by Ricardo et al., the average time from presentation of HH to death was short (368 days), and the outcome was extremely poor in these groups except for those undergoing trans-jugular intrahepatic portosystemic shunt or LT [14]. Moreover, they also proved sepsis was the most common cause of mortality (37%) [14]. This result correlates with our findings and indicates that HH is a marker of ESLD associated with high infection and mortality rate. Thus, drainage of HH and early LT are recommended to prevent complication [14].

Technical factors may have been related to septic complications after GI procedures. Several studies concluded that factors have not been associated with bacteremia, such as the underlying bowel pathology, duration of the procedure, or performance of endoscopic biopsies or polypectomy [26]. In our study, neither biopsy nor polypectomy in upper and lower GI endoscopy induced sepsis (Table 5).

Our patients received combined EGD and CFS, and it could not be differentiated whether sepsis came from the upper or lower GI tracts. In prospective studies, upper and lower GI endoscopy was associated with bacteremia rates of 4%, and the observed bacteremia usually was short lived and not caused infectious adverse events [27, 28]. Although reports of infectious sequelae are rare, there are several case reports of bacteremia and mortality following colonoscopy in cirrhotic patients [10, 11, 29]. The prospective study by Josep et al. [30] concluded that lower intestinal endoscopy does not induce bacteremia in cirrhotic patients in the absence of GI bleeding and recommended against the routine antibiotic prophylaxis. That study recruited 58 cirrhotic patients with 28 and 21 patients categorized as Child–Pugh B and C status, respectively. Compared with the previous study, our study enrolled more decompensated liver cirrhotic patients, including 259 Child–Pugh B and 159 Child–Pugh C patients out of a total of 642 LT candidates. Moreover, all of our patients stayed in the hospital after the procedure for close monitoring of their clinical condition and to obtain a more precise record of vital signs.

For screening, adequate bowel preparation is required to ensure colonic cleaning. A solution with PEG is safer than oral sodium phosphate for low risk of renal injury, especially in cirrhotic patients who have the potential for hepatorenal syndrome. Liver cirrhosis was a strong predictor for poor bowel preparation, probably due to ascites and general weakness, which make patients unable to tolerate too much fluid intake [31]. Therefore, we used a low volume of PEG (2 L) rather than a standard volume (4 L) for colon cleaning. Although the cause of post endoscopic sepsis was unknown in our study, bowel preparation does not enhance bacterial translocation or even sepsis according to several studies [32].

The risk of pulmonary aspiration during sedative EGD has been reported and may be up to 3.9% [33]. Concerning this potential adverse event, chest X-ray was performed for every sepsis patient in our study, but none of them was diagnosed with aspiration pneumonia. This may be explained by the minimal residual gastric food after fasting for more than eight hours and the stand-by of specialized anesthesia doctors and assistants providing sufficient sedation and continuous saliva suction.

According to the ASGE guidelines for GI procedures from 2015, prophylactic antibiotic administration is recommended for percutaneous endoscopic gastrostomy, endoscopic ultrasound-guided fine needle aspiration, endoscopic retrograde cholangio-pancreatography, and patients with cirrhosis admitted for GI bleeding, and which should receive antibiotic therapy with third-generation cephalosporin [9]. Based on this, we used third-generation cephalosporin (ceftazidime, ceftriaxone, or flomoxef) as a prophylactic antibiotic agent. Flomoxef was used more often in our practice for the additional coverage of anaerobic infection compared with ceftazidime and ceftriaxone.

The four positive blood cultures in the experience cohort indicated *Micrococcus, S. aureus, E. coli,* and *A. sobria* bacteremia. In the validation cohort with overall antibiotic prophylaxis, there were only four cases of post-endoscopic sepsis, and the culture revealed negative results. An association between bacteremia and antibiotics was also evident in our study (0% vs. 1.3% in the groups with and without antibiotic prophylaxis, respectively; *P* = 0.032).

### Limitations of the study

This study had several limitations. Firstly, the retrospective data may have impacted the identification of some confounding factors. Secondly, the study was conducted in a single medical center, and sepsis associated with GI endoscopy may vary in different hospitals. Thirdly, because EGD and CFS were performed together, it was difficult to differentiate sepsis coming from single and combined procedures. Finally, the study recruited only 642 patients. Therefore, a large, prospective, randomized, controlled trial is required to study the incidence of sepsis after GI endoscopy in cirrhotic patients.

## Conclusions

The literature points out that infectious complications are uncommon sequelae of GI endoscopic procedures in the general population. In cirrhotic patients, however, sepsis may happen within two days after elective upper and lower GI endoscopy. Our findings indicate GI endoscopy induces sepsis in cirrhotic patients in the absence of acute bleeding, especially among patients with HH and Child–Pugh B and C decompensated liver cirrhosis. Drainage of HH and antibiotic prophylaxis prior to endoscopy in advanced cirrhotic patients could reduce the occurrence of sepsis.

## Data Availability

The dataset supporting the conclusions of this article are included within the article and files. The dataset used during the current study are available from the corresponding author on reasonable request.
